# Prevalence and Antimicrobial Susceptibility Profile of *E. coli* O157:H7 in Fish Collected From Lake Ziway, Ethiopia

**DOI:** 10.1155/bmri/5097078

**Published:** 2026-06-10

**Authors:** Assaye Desta Amare, Fufa Abunna Kurra, Fikru Regassa Gari

**Affiliations:** ^1^ Department of Veterinary Medicine, School of Veterinary Medicine, Bahir Dar University, Bahir Dar, Ethiopia, bdu.edu.et; ^2^ Department of Veterinary Epidemiology and Public Health, College of Veterinary Medicine and Agriculture, Addis Ababa University, Bishoftu, Ethiopia, aau.edu.et; ^3^ Department of Biomedical Sciences, Colleges of Veterinary Medicine and Agriculture, Addis Ababa University, Bishoftu, Ethiopia, aau.edu.et

**Keywords:** antimicrobial resistance, *Escherichia coli* O157:H7, Ethiopia, fish, Lake Ziway, latex agglutination test

## Abstract

**Background:**

Fisheries play a significant role in food security, livelihood, and source of income in developing countries. Despite this, fish can spread infections.

**Methods:**

A cross‐sectional study with a simple random sampling approach was conducted to determine the prevalence and antibiotic susceptibility profile of *E. coli* or O157:H7 isolate from fish and fish handling equipment (knife) in Lake Ziway, Batu town, Ethiopia. To isolate and identify *E. coli* O157:H7, a total of 220 samples were collected by simple random sampling approach. Muscle and skin samples were collected from each 120 fish comprising Nile tilapia, common carp, and catfish obtained from landing sites and cold storage establishments of restaurants. One hundred samples were collected from fish processing knives and the hands of fish handlers. All the microbial identification and isolation procedures were conducted based on selective culture media isolations. Antimicrobial susceptibility was conducted based on Kirby–Bauer disk diffusion protocols.

**Findings:**

Overall prevalence of O157:H7 was found (4.5%). *E. coli* O157:H7 was detected in two (3.3%) of the total 100 fish handling equipment and eight (6.7%) of the total 120 fish samples. *E. coli* O157:H7 was isolated at 5.7% and 8% prevalence in frozen fish collected from restaurant cold storage and fresh fish collected immediately from the landing site. Antimicrobial susceptibility test of *E. coli* O157:H7 isolates for kanamycin (30 ug), tetracycline (30 ug), cefoxitin (30 ug), erythromycin (20 ug), streptomycin (10 ug), sulfamethoxazole (25 ug), and ampicillin (10 ug) showed resistance to most antibiotics. The results indicate that *E. coli* O157:H7 was isolated from 10 samples from fish and fish handling equipment in this study. In addition, isolates were found resistant to penicillin and ampicillin. Kanamycin, tetracycline, and cefoxitin were found effective in inhibiting the growth of all of the isolates.

**Conclusion:**

The presence of *E. coli* O157:H7 isolates and its resistance to drugs pose significant economic and public health challenges. Thus, it requires implementing interventions like freezing and proper fish handling practices to minimize the level of contamination of fish in Ethiopia.

## 1. Introduction

Fisheries play a significant role in food security, livelihood, a source of income, and social development in developing countries [1]. Ethiopia′s freshwater capture fisheries have the capacity to yield 51,481 tons of fish per year [2]. From this amount of total annual yield, Lake Ziway contributes 2300 tons yearly ([3]). Although fish are a healthy source of protein, they can also spread diseases caused by pathogenic microorganisms they may harbor [1]. *E. coli* is a well‐known foodborne pathogen that is commonly detected in contaminated food and water and deteriorating fish quality. It is a natural part of the gut microbiota in both humans and healthy animals [2].

*E. coli* O157:H7 is a gram‐negative, cylindrical, nonsporing bacterium [4]. Genes in *E. coli* O157:H7 enable the production of toxins called verotoxins or Shiga‐like toxins (Stxs), which can result in hemolytic‐uremic syndrome, hemorrhagic colitis, and in extreme instances, even death [5]. According to [6], *E. coli* O157:H7 has been shown to have an infectious dose as low as 10 cells, which is lower than that of the majority of other Enterobacteriaceae. Ruminants are the primary reservoirs that passively release and spread *E. coli* O157 without producing symptoms, according to [7]. Additionally, ruminants frequently harbor *E. coli* O157:H7 as hosts. Few attempts have been made to discover *E. coli* O157:H7 in fish that have been farmed in Ethiopia. In Ethiopia, stool samples from Bishoftu Hospital; minced beef from an Addis Ababa supermarket; cattle, sheep, and goat meat from export and municipal abattoirs in Debre Zeit and Modjo; and beef, sheep, and goat meat from Dire Dawa, have all been reported to contain *E.* coli O157:H7 [8]. These reports indicated the presence of *E.coli* O175:H7 in Ethiopia. Studies have demonstrated the potential for resistant bacterial strains to proliferate among humans, animals, animal derivatives, and the environment [9].

Antibiotic‐resistant foodborne microbes may be more dangerous to human health [2]. Consequently, antibiotic susceptibility testing is required to attain a better response [4]. *E. coli* O157:H7 is widely recognized as a zoonotic pathogen, most commonly linked to contaminated meat and dairy products. In contrast, its presence in fish and aquatic ecosystems has received limited attention, particularly in Lake Ziway, Ethiopia. Lake Ziway is a key inland water body with considerable public health and economic relevance, yet no published data are available on the prevalence or antimicrobial resistance profile of *E. coli* O157:H7 in fish from this lake. Investigating this issue is therefore essential to support evidence‐based food safety interventions and antimicrobial resistance surveillance in the region [1, 10, 11]. Thus, this study is aimed at determining the prevalence and antimicrobial susceptibility profile of *E. coli* O157:H7 in fish collected from Lake Ziway in Ethiopia.

## 2. Materials and Methods

### 2.1. Ethical approval

The study was conducted according to the guidelines of Addis Ababa University for animal research ethics (Ethiopia).

### 2.2. Description of the Study Area

**Figure 1:**
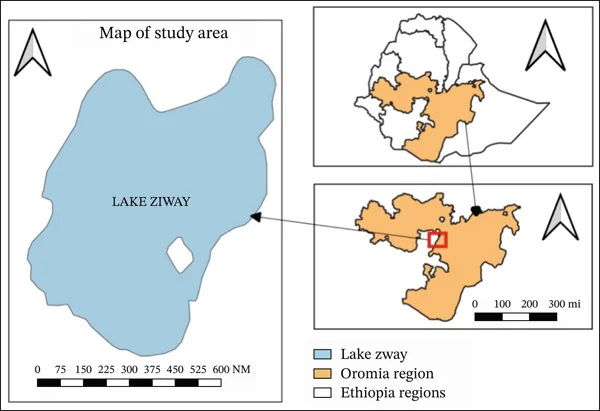
Map of Lake Ziway, Ethiopia study area.

Lake Ziway is situated (Figure 1) 175 km southeast of Addis Ababa, the capital of Ethiopia, in the Great East African Rift Valley Lakes region. It has an average depth of 2.5 m, a total area of 434 km^2^, and is located at 1636 meters above sea level (MASL). The lake watershed spans approximately 7300 km^2^ and is located between latitudes 7°15 ^′^N and 8°30 ^′^N and longitudes 38°E and 39°30 ^′^E. The climate is classified as semiarid to subhumid, with an average temperature of 25°C and 650 mm of precipitation overall. Batu is a town and distinct woreda (administrative structure) located in the central Ethiopian districts of Adami Tulu Jiddokombolcha (ATJK). The town of Batu is located on the lake′s western bank. It has been expanding quickly in recent years [12].

### 2.3. Study Population

All fishes originated from Lake Ziway namely; Niltilapia (*Tilapia nilotica*), Common carp (*Clarius garpinus*), and catfish species of fish were populations of this study. In addition to this, swab samples were also taken from the hands of fish handling personnel before handling fish, and knife swabs before they use knives for degutting and filleting. From restaurants, only fish samples were taken.

### 2.4. Study Design

In order to isolate, identify and determine the antimicrobial susceptibility profile of *E. coli* O157:H7 isolates from the fish and fish handling environment of Lake Ziway in Batu Town; a cross‐sectional study was conducted from November 2020 to April 2021. A total of 220 fish and environmental samples were randomly collected from collecting centers, cafeterias, and cold storage establishments located in Batu town.

### 2.5. Sample Size Determination

The predicted prevalence of *E. coli* (15.10%) by [13] in and around Bishoftu lakes was used to establish the required sample size for this investigation. This is because Lake Bishoftu and Lake Ziway are rift valley lakes with similar agroclimatic conditions. This is because using prevalence in Bishoftu Lakes was essential as there are no prior statistics provided in the study area. By applying the method from [14] and assuming a 95% confidence level and a 5% required level of precision, establishments according to the following formula.

n=1.962 Pexp 1−Pexpd.

In this case, d refers to desired absolute precision at 95% Confidence level, n refers to the required sample size, q refers to 1‐p, and Pexp refers to expected prevalence (expected prevalence of *E. coli*, 15%, according to [14]. The aforesaid formula indicated that 195.89~196 samples were needed; nevertheless, in order to improve the degree of precision, a total of 220 samples were gathered for this investigation.

### 2.6. Sampling Strategy

Different sampling techniques were applied during the study period to select the major fish collection centers and cold storage in Batu Town. Korokonch, Menafesha, and Bochesa were fish collecting centers selected purposively; based on fish collection potential and accessibility. Other small landing sites were bringing fish to these major landing sites. Batches of fish from each species were selected randomly from cold storage, the establishments were randomly selected among the available fish traders, and batches of frozen fish were also randomly selected from their cold chains. The adult fish catch from Lake Ziway consists of almost exclusively Nile tilapia (*Oreochromis Niloticus*), catfish (*Clarias Garpinus),* and Crucian carp (*Caracius gracious*) [11]. All these species of fish were included in this study and they were randomly selected. Besides, 100 swab samples were collected from fish handling environments such as from fish degutting knives and hand swabs of fish handlers.

### 2.7. Source of Fish, Sample Collection Procedures, Handling, and Transportation

The source of the fish used in the study was collected from fishers operating in Lake Ziway. Whole fish (skin) swab filleted, fish muscle (tissue), knife and hand swab procedures (29). Fish samples were identified and placed in sterile plastic bags; ice packed in an ice box (0°C) and transferred to the Microbiology Laboratory, College of Veterinary Medicine and Agriculture, Addis Ababa University. After arrival, the skin surface and muscles of each fish were swabbed aseptically as described for bacteriological isolation, identification, and antimicrobial susceptibility testing [4]. Before processing, the frozen samples were allowed to thaw at room temperature [15]. The forceps, scalpel blades, and other instruments used were sterilized with flame and disinfected with alcohol before working.

Using sterile swabs wet in sterile buffered peptone water (1%), environmental samples were gathered. Immediately before fish processing began, swabbing the fish operators′ hands and knives, and then submerging them in test tubes filled with sterile buffered peptone water (1%). The tubes were labeled with name, source, and date of collection. The tubes were put on ice packed and transferred (0°C) to the laboratory.

### 2.8. Sample Preparation, Isolation and Identification of *E. coli* O157:H7

All the isolation and identification procedures of the organism were performed based on ISO 6887‐3:2017 recommendation for microbiological analysis of fish samples (29). Essentially, the samples were initially pre‐enriched in 1% buffered peptone water and then incubated at 37°C for 24 h. ON MacConkey′s agar (Oxoid, Canada), a loopful was streaked with a sterile loop, and the mixture was then incubated for 24 hours at 37°C. Gram stain was used to color typical pink colonies on MacConkey′s agar. The bacterial cultures were then plated onto eosin‐methylene‐blue agar (CDH, India) and their color and physical characteristics were recorded [16].

To verify the colonies with a metallic sheen on EMB agar, a series of biochemical tests including the indole test, methyl red test, Voges‐Proskauer test, citrate utilization tests (IMVIC tests), HS2 production test, catalase test, and sugar fermentation were conducted using standard procedures to confirm the presence of *E. coli*′s characteristic features [16].

The *E. coli* isolates from the foregoing process were subcultured in 1% sorbitol MacConkey′s agar and kept at 37°C for a full day. Among these isolates, the colonies that were colorless or pale and did not ferment sorbitol were classified as *E. Coli* O157:H7 strains, whereas the colonies that were pinkish in color and did ferment sorbitol were classified as non‐E*. Coli* O157:H7 strains. The bacterial colony was picked and subjected to a slide agglutination test using an *E. coli* O157 latex kit (Oxoid Ltd., Hampshire, United Kingdom). A drop of test latex and sterile saline water was dispensed into the reaction card separately. Up to five presumptive *E. coli* O157:H7 colonies were picked by lightly touching the center of the colony with a sterile inoculating needle. The picked colonies were thoroughly emulsified with the saline on latex card and then finally with the test latex reagent. The formation of agglutination within 1 min was regarded as positive as stated by [4].

### 2.9. Antimicrobial Susceptibility Testing of *E. coli O157*:*H7* Isolates

The susceptibility pattern of *E. coli* O157:H7 isolates to 10 commonly used antibiotics within the research area was determined using the standard disc diffusion technique protocol as outlined in [17]. Antimicrobial agent concentrations are listed in Table 1. A meter ruler was used to measure the zone of inhibition to the closest millimeter, and the Clinical Laboratory Standard Institute′s recommended criteria were followed for interpretation [17]. The antimicrobial panel was selected based on the Clinical and Laboratory Standards Institute (CLSI, 2012) guidelines. The panel focused on agents commonly used in human therapy for invasive *E. coli* infections and those frequently applied in aquaculture.

**Table 1:** Antimicrobial′s test used for susceptibly of *E. coli* O157:H7 isolated from fish, knife swabs, and hand swabs from Lake Ziway, Ethiopia.

Antimicrobial disc	Code	Concentration (ug)	mm		
			Resistant	Intermediate	Susceptible
Ampicillin	AMP	10 ug	13	14–16	17
Cefoxitin	FOX	30 ug	14	15–17	18
Penicillin G	P	10 ug	28	NA	29
Erythromycin	ER	20 ug	15	16–20	21
Tetracycline	TE	30 ug	11	12–14	15
Streptomycin	S	10 ug	11	12–14	15
Sulfamethoxazole	SXT	25 ug	10	11–15	16
Kanamycin	K	30 ug	13	14–17	18

*Note:* Resistant, if less than or equal to the diameter of the inhibition zone. Susceptible, if greater than or equal to the diameter of the inhibition zone.

Abbreviations: IU, International unit; mm, diameter of zone of inhibition (CLSI, 2012); ug, Microgram.

### 2.10. Statistical Analysis

After being entered into an Excel spreadsheet application, the acquired data was examined using Stata Version 14. The data was summarized using descriptive statistics in terms of percentages and frequencies. To display the data, there were tables, charts, and graphs. With the use of the chi‐square (*X* ^2^) test and *p* value, the relationship between the various components was examined. The significant level was established using a 95% confidence interval and *p* < 0.05 confidence levels [18].

## 3. Results

### 3.1. Isolation and Identification of E. coli O157:H7

**Figure 2:**
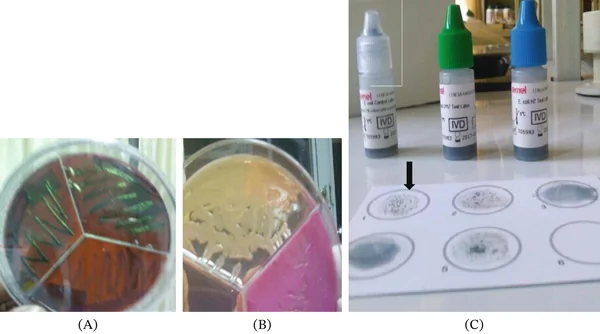
Isolation and identification of E*. coli* and *E. coli* O157:H7 from fish samples. (A) Colony characteristics of *E. coli* from fish on EMB agar; (B) *E. coli* O157:H7: on sorbitol‐MacConkey; (C) *E. coli* latex agglutination test procedure, “arrow” indicates agglutination with the clear background within 1 min which showed a positive result.

*E. coli* colonies were found green metallic sheen colonies on eosin methylene blue agar as indicated in Figure 2A. *E. coli* 157:H7 was described as pale colorless colonies on sorbitol MacConkey agar as indicated in Figure 2B; *E. coli* 157:H7 isolates were confirmed using *E. coli* latex agglutination test procedure, where agglutination with a clear background within 1 min showed positive result (Figure 2C).

Regarding sources of fish samples, *E. coli* was found at a higher percentage (47.1%) in fresh fish collected from fish collecting centers than from frozen fish 16 (32%). Whereas *E. coli* O157:H7 was isolated at 8% in frozen fish and 5.7% in fish. Regarding fish species, the existing difference for isolation of *E. coli* and *E. coli* O157:H7 was not found statistically significant.

### 3.2. Antimicrobial Susceptibility Testing Profile

**Figure 3:**
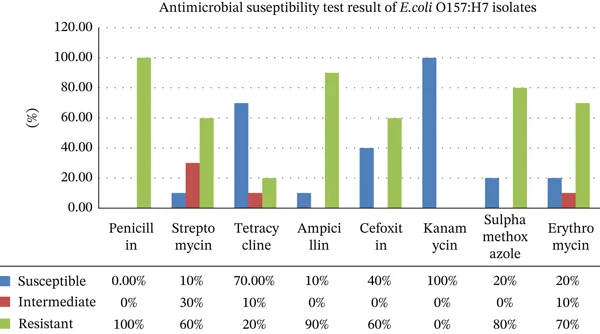
Antibiotic susceptibility profile of *E. coli* O157:H7 isolated from skin and muscle samples of Nile tilapia fish from Lake Ziway, Ethiopia in 2020 GC.

Among the 10 *E. coli* O157:H7 isolates examined, the antimicrobial susceptibility testing results displayed in (Figure. 3) indicate that all isolates were susceptible to kanamycin (100%). Additionally, seven isolates (70%) were susceptible to tetracycline, four isolates (40%) to cefoxitin, two isolates (20%) to erythromycin, one isolate (10%) to streptomycin, two isolates (20%) to sulfamethoxazole, and one isolate (10%) to ampicillin. However, all isolates exhibited resistance to penicillin G (100%). Furthermore, for tetracycline, erythromycin, and streptomycin, one (10%), one (10%), and three isolates (30%) were presented intermediate resistance, respectively (Figure 3).

## 4. Discussion

This study clearly shows that 10 (4.5%) of the *E. coli* O157:H7 isolates were detected in fish and environmental samples that were gathered and examined during the study period. Overall, the prevalence of *E. coli* infection in fish reported in these findings (35.5%) was related to findings from prior investigations from Bishoftu, Ethiopia (15.01%) by [13] and in other countries, by [19, 20].

However, it was lower than the report found by [21], which found 100% of the fish were contaminated with *E. coli*. Variations in sample size, sample type, sample laboratory processes, and sanitary conditions employed are some of the sampling strategies that may have contributed to the resulting discrepancies between this finding and with findings of other researchers.

The prevalence of *E. coli* O157:H7 10 (4.5%) in the research was comparable to that of [22]. Related findings were reported from Lake Hayq and the Tekeze Dam in Ethiopia by [1] and from Scotland by [23].

However, these findings contradict those of a previous study [24], which did not detect any *E. coli* O157 in the fish in India. This finding was lower compared to the findings of [25] who reported a 10% prevalence of *E. coli* O157:H7 in raw beef, mutton, and chevon collected from the Addis Ababa Abattoir Enterprise and selected retail shops in Addis Ababa, Ethiopia. The most likely source of *E. coli* O157:H7 infection in fish is environmental variables that occur throughout the fish harvesting and production process. Uncontrolled environmental management in the study area has resulted in pollution of lake [10]. The presence of *E. coli* O157:H7 at 2 (3.3%) in the hand swab samples (Table 2) has suggested that fish workers were not properly washing their hands. Additionally, it raises the possibility that the fish could become infected while being handled, degutted, filleted, and transported [26].

**Table 2:** Occurrence of *E. coli* O157:H7 in different types of swab samples collected from fish and fish handling environment from Lake Ziway, Ethiopia from November 2020 to April 2021.

Type of samples	Isolates						
	*E. coli*				*E. coli* O157:H7		
	Number examined	Number (%) positive	*X* ^2^	*p*	Number (%) positive	*X* ^2^	*p*
Fish	120	49 (40.8%)	1	0.047	8 (6.7%)	2	0.98
HS	60	19 (31.7%)			2 (3.3%)		
KS	40	9 (22.5%)			—		
**Total**	**220**	**77 (35.5%)**			**10 (4.5%)**		

Abbreviations: HS, hand swab; KS, knife swab; *X* ^2^, chi‐square.

The body surface of the fish had significantly greater contamination with *E. coli* 46 (38.3%) than the muscle sample 12 (10%) of all the fish that were investigated (Table 3). *E. coli* O157:H7 was more common in six skin samples than in muscle samples [10]. The results also confirm previous findings that one of the most important and prevalent bacteria on fish body surfaces is *E. coli* [10, 27]. This finding implies that the skin′s close contact with water and other environmental pollutants, which contain a high concentration of dangerous germs, may be the cause of this.

**Table 3:** Isolation of *E. coli* and *E. coli* O157:H7 in fish regarding different risk factors from Lake Ziway, Ethiopia from November 2020 to April 2021.

		Isolates						
		*E. coli*				*E. coli* O157:H7		
Risk factors		N	Number (%) positive	*X* ^2^	*p*	Number (%) positive	*X* ^2^	*p*
Species	Nile tilapia	60	26(43. %)	0.86	0.65	4 (6.7%)	0.54	0.76
	C. Carp	40	14(35%)			2 (5%)		
	Catfish	20	9(45%)			2 (10%)		
Source	Landing	70	33(47.1%)	2.77	0.96	4 (5.7%)	0.24	0.874
	Storage	50	16 (32%)			4 (8%)		
Organs of fish	Skin	120	46(38.3)	26	0.001	6 (5%)	2	0.15
	Muscle	120	12(10.0%)			2 (1.7%)		
Total		**120**	**49(40.8%)**			**8 (6.7%)**		

Abbreviations: C. carp, common carp; N, total no of fishes examined; *X* ^2^, chi‐square.

The difference that was observed could have resulted from inadequate cleanliness during handling and processing; skin is conducive to rapid bacterial growth and environmental adaption, which is in line with research findings [28].

As the skin has direct contact with the environment and can easily come into contact with numerous pathogenic or nonpathogenic *E. coli*, the fish muscle can become contaminated with various bacteria during harvesting. This is one of the scientific reasons for the existence of *E. coli* O157:H7 in muscle, which is normally free of pathogenic microorganisms [27, 29].

Although *E. coli* O157:H7 was isolated in fresh fish from landing centers at a rate of 5.7% and frozen fish from cold storages at a rate of 8%, the difference was deemed statistically insignificant for fish origin. According to [30, 31], isolates discovered in frozen fish indicated contamination, which might have been caused by handling the fish after harvest, inadequate storage, and bad ice.

The observed variation in prevalence of *E. coli* O157:H7 fish species was determined to be statistically insignificant. This demonstrated that there is no possible possibility of increased frequency contamination due to species differences.

**Figure 4:**
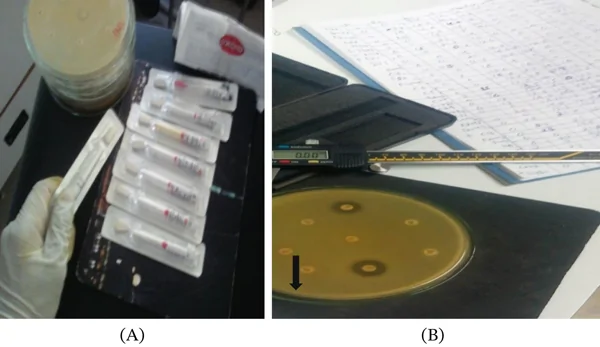
Antimicrobial susceptibility profile of *E. coli* O157:H7 isolated from skin and muscle samples of fish. (A) Antimicrobial susceptibility pattern of *E. coli* O157:H7 isolates from skin and muscle samples of fish. (B) Arrow representing microinhibition zone for selected antibiotics test.

Study in Ethiopia had observed that *E. Coli* O157:H7 isolates from both animal and human sources exhibited resistance to antibiotics [32]. Every single one of the 10 isolates in this investigation (Figure 4) exhibited strong susceptibility to kanamycin (30 ug) with tetracycline (30 ug) coming in second. This indicates that in our study area, these antibiotics continue to be the preferred medication for treating infections caused by *E. coli* O157:H7. This result is also consistent with the research of [32, 33]. The pathogen′s development of resistant gene code, which is connected to the isolates′ emerging and reemerging traits in relation to different agroecologies, may be the cause of this variation [34].

Among 10 *E. Coli* O157:H7 isolates resistance to ampicillin, erythromycin, and penicillin G were found the most often detected resistance profile. A similar finding was reported by Shitandi and Stemejo (2001). The current investigation indicated that *E. Coli* O157:H7 had a significant level of resistance to penicillin G and ampicillin. Resistance to erythromycin and penicillin G these agents represents intrinsic mechanisms and should not be interpreted as acquired antimicrobial resistance. This might be a result of the drug′s widespread availability in the research area for both routine prophylaxis and treatment. Although the Gram‐negative bacterium *E. coli* O157:H7 has the beta‐lactamase enzyme, penicillin G and ampicillin are beta‐lactam antibiotics.

This enzyme has the ability to render beta‐lactam antibiotics inactive. This result indicated that *E. coli* O157:H7 frequently exhibits high erythromycin resistance, which is also in line with reports from Saudi Arabia [33, 35]. This study also discovered cefoxitin resistance, which is considered exceptional; comparable findings were reported from Canada [36]. Seven of the isolates from the eight drugs exhibited a particular pattern of resistance, with the resistance rate ranging from 10% to 100%.

Overuse of antibiotics during treatment of animals could lead to the emergence of multiple drug resistance. Because they are frequently carriers of the bacterium, ruminants or livestock can release *E. coli* O157:H7 into the environment through their excrement for an extended period of time [7].

### 4.1. Study Limitation

The study was limited by the low number of confirmed *E. coli* O157:H7 isolates (*n* = 10). As the sample size calculation was based on generic *E. coli* prevalence, the study was underpowered to perform extensive inferential statistical analysis on the specific risk factors for O157:H7 infection. Therefore, the findings regarding this serotype should be interpreted as descriptive and preliminary.

## 5. Conclusion

In this investigation, the overall prevalence of *E. coli* O157:H7 was 10 (4.5%). The fish may be contaminated with *E. coli* O157:H7 as a result of poor storage, unsanitary postharvest handling procedures, and tainted water. The potential concern to consumer health is highlighted by the existence of resistant isolates and the isolation of *E. coli* O157:H7 from both fresh and frozen fish. Nearly every isolate exhibited a high degree of ampicillin and penicillin G resistance. Although the isolation of *E. coli* O157:H7 was limited (*n* = 10), preventing a broad assessment of regional AMR trends, the identification of resistance to cefoxitin, erythromycin, streptomycin, sulfamethoxazole, and ampicillin was performed. These isolates highlights a potential public health risk that warrants further surveillance with larger sample sizes. In the study area, the preferred medications for *E. coli* O157:H7 fish disease with these bacterial illnesses were tetracycline and kanamycin, with kanamycin being the most recommended. Overall, the investigation discovered a significant incidence of *E. coli* O157:H7 contamination and a safety gap in fish handling. Regular testing for antimicrobial susceptibility is necessary to determine whether antibiotics are effective and to address the problem of drug resistance emerging against commonly used drugs for fish.

In the present study, *E. coli* O157:H7 was found in hand swabs, knife swabs, and fish samples. Therefore, during fish handling and processing, fish may be exposed to *E. coli* O157:H7. To identify the key source of *E. coli* O157:H7, more research on lake water, ice, and other fish processing equipment is needed. Additionally, the bacteria in lake water should be separated and identified.

## Author Contributions

Assaye Desta Amare contributed in writing up the original research, data collection, and methodology and statistical data analysis. Fufa Abunna Kurra and Fikru Regassa Gari contributed in supervision, software, statistical data analysis, and methodology.

## Funding

No funding was received for this manuscript.

## Ethics Statement

Ethical issues (including plagiarism, consent to publish, misconduct, data fabrication and/or falsification, double publication and/or submission, and redundancy) have been checked and compiled by the authors.

## Consent

The authors have nothing to report.

## Conflicts of Interest

The authors declare no conflicts of interest.

## Data Availability

Data available on request due to privacy or ethical restriction.
